# Mobile stroke unit in the UK healthcare system: avoidance of unnecessary accident and emergency admissions

**DOI:** 10.29045/14784726.2021.3.5.4.64

**Published:** 2021-03-01

**Authors:** Daniel Phillips, Iris Q. Grunwald, Silke Walter, Klaus Faßbender

**Affiliations:** East of England Ambulance Service NHS Trust; Southend University Hospital NHS Foundation Trust; Anglia Ruskin University; University of Dundee; Anglia Ruskin University; Saarland University Medical Center; Saarland University Medical Center

## Abstract

**Aims:**

The aim of the study was to explore the benefit of a mobile stroke unit (MSU) in the UK National Health Service (NHS) for reduction of hospital admissions.

**Methods:**

Prospective cohort audit observation with dispatch of the MSU in the East of England Ambulance Service was conducted. Emergency patients categorised as code stroke and headache were included from 5 June to 18 December 2018. Rate of avoided admission to the accident and emergency (A&E) department, rate of admission directly to target ward and stroke management metrics were assessed.

**Results:**

In 116 MSU-treated patients, the following diagnoses were made: acute stroke, n = 33 (28.4%); transient ischaemic attacks, n = 13 (11.2%); stroke mimics, n = 32 (27.6%); and other conditions, n = 38 (32.8%). Pre-hospital thrombolysis was administered to eight of 28 (28.6%) ischaemic stroke patients. Pre-hospital diagnosis avoided hospital admission for 29 (25.0%) patients. As hospital treatment was indicated, 35 (30.2%) patients were directly triaged to the stroke unit, one patient (0.9%) even directly to the catheter laboratory. Thus, only 50 (43.1%) patients required transfer to the A&E department. Moreover, the MSU enabled thrombolysis with a median dispatch-to-needle time of 42 mins (interquartile range, 40–60).

**Conclusion:**

This first deployment of an MSU in the UK NHS demonstrated improved triage decision-making for or against hospital admission and admission to the appropriate target ward, thereby reducing pressure on strained A&E departments.

